# Time Is Kidney: A Case Study and Literature Review of Bilateral Renal Compartment Syndrome After Blunt Trauma, a Rare Complication

**DOI:** 10.3390/jcm15062466

**Published:** 2026-03-23

**Authors:** Luis Fernandez, Ahmad Jalal Kanawati, Mohamed Abdelgawad, Diana Wu, Brittany Wagner, Andrew Navetta, Marc Mathews, Sarah Kamel, Andrew Armanyous, David Villareal

**Affiliations:** 1 Department of General Surgery, School of Medicine, University of Texas at Tyler, Tyler, TX 75799, USA; 2 College of Medicine, Ajman University, Ajman P.O. Box 346, United Arab Emirates; 3 Department of Surgical Oncology, Allegheny Health Network, Pittsburgh, PA 15212, USA; 4 College of Medicine, University of Arizona, Tucson, AZ 85752, USA; 5 College of Medicine, University of Queensland, Brisbane 4072, Australia; 6 University Campus, Avalon University School of Medicine, Willemstad P.O. Box 480, Curacao

**Keywords:** renal compartment syndrome, perinephric hematoma, subcapsular hematoma, abdominal compartment syndrome, blunt trauma, renal transplant, renal failure

## Abstract

**Background:** Acute compartment syndrome (ACS), a condition characterized by elevated pressure within an enclosed compartment, leads to ischemia and organ failure, and is hence a surgical emergency. Renal compartment syndrome (RCS) is a disease in which there is an increase in the pressure within the native kidney’s compartment due to peri-renal or subcapsular fluid collection, causing acute kidney injury. To our knowledge, the diagnosis of bilateral traumatic renal compartment syndrome (BTRCS) due to trauma has not been previously described in the literature. **Case Presentation:** The patient is a 20-year-old female presenting as a case of blunt trauma due to a severe motor vehicle collision. Initially, investigations showed multiple injuries, including a femur fracture that was managed accordingly. Postoperatively, she remained stable with no signs of complications. However, after 10 days, she began complaining of abdominal pain. Further workup revealed an acute drop in hemoglobin, elevated serum creatinine, and bilateral perinephric hematomas. BTRCS was diagnosed and was surgically managed by open laparotomy and bilateral capsulotomy, with the return of robust urine production. The patient recovered successfully and was consequently discharged. **Conclusions:** This paper reports a case of renal compartment syndrome that was diagnosed and treated appropriately. Doppler ultrasound and CT scan, along with renal function tests, are the investigations of choice. Although there can be a role for conservative management, open surgical decompression remains the definitive treatment in patients with progressive renal dysfunction. To our knowledge, this represents the first reported case applying the term “bilateral traumatic renal compartment syndrome (BTRCS)” involving native kidneys following blunt trauma, successfully treated with bilateral surgical decompression and rapid physiological recovery.

## 1. Introduction

Acute compartment syndrome (ACS) is defined by increased pressure within a closed compartment, resulting in impaired local circulation, causing tissue ischemia and organ failure. Without proper treatment and decompression, it can lead to severe physiologic complications and is considered a surgical emergency [1].

The diagnosis of extremity compartment syndrome involves a careful clinical examination and the measurement of the involved compartment’s pressure, but both have limitations [2]. The World Society of Abdominal Compartment Syndrome released a classification for intra-abdominal hypertension, which is defined as a sustained intra-abdominal pressure greater than 20 mm Hg associated with new organ dysfunction or failure [3]. Compartment syndrome of the abdomen and extremities in the setting of trauma is a well-established diagnosis with well-published diagnostic algorithms to assist in establishing this diagnosis [4].

Isolated renal allograft compartment syndrome (RACS) has been documented in the transplant literature, occurring in the post-transplant setting of the allograft, but remains a rare occurrence (~2%) [5]. RACS is characterized by increased compartmental pressure over 15 to 20 mm Hg of the iliac fossa site of the transplanted kidney, which leads to diminished perfusion to the graft, causing ischemia and graft loss. Treatment involves immediate surgical decompression and a tension-free closure to affect graft recovery [6]. Similarly, renal compartment syndrome can happen within the native kidneys in their normal retroperitoneal anatomic location.

In this report, we present a case of a 20-year-old female who developed bilateral renal compartment syndrome in the setting of blunt abdominal trauma.

### Key Learning Points

- Bilateral traumatic renal compartment syndrome is an extremely rare but potentially reversible cause of acute kidney injury after blunt trauma.
- The diagnostic triad includes acute kidney injury, perinephric or subcapsular collections on imaging, and reduced renal perfusion on Doppler ultrasound.
- Early recognition is critical as surgical decompression via renal capsulotomy can rapidly restore renal perfusion and urine output.
- Trauma surgeons should maintain a high index of suspicion for renal compartment syndrome in patients with worsening renal function and expanding perinephric hematomas.

## 2. Case Presentation

**Figure 1:**
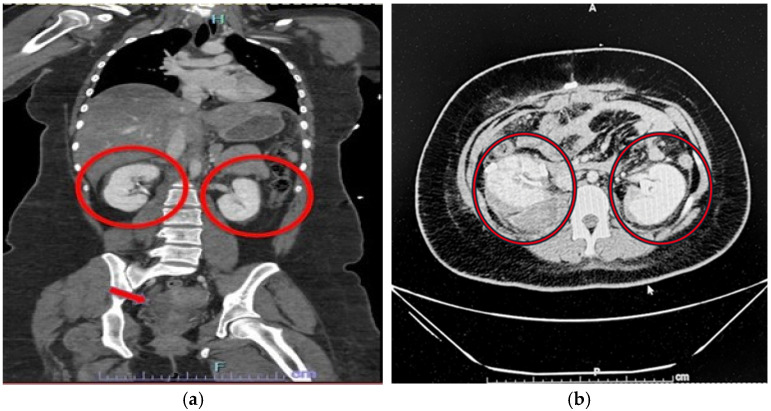
Index CTAPiv coronal (**a**) and axial (**b**) images demonstrate a grade 2 splenic laceration, right adrenal contusion, small bilateral perinephric hematomas (circles), and a small volume hemoperitoneum (arrow).

A 20-year-old female arrived at UT Tyler Level I Trauma Center as a level 2 trauma activation transported via Air EMS. She presented as a restrained rear-seat passenger involved in a severe two-vehicle MVC with prolonged extrication. Upon arrival, she had a Glasgow Coma Scale of 15 with stable vital signs. She was fully immobilized with an extended spinal board and a C-collar, and her left leg was placed in a traction splint for a midshaft femur deformity. A computerized tomographic scan of the abdomen and pelvis with intravenous contrast (CTAPiv) demonstrated additional injuries, including a grade 2 splenic laceration, a right adrenal contusion, small bilateral perinephric hematomas (Figure 1), a small volume hemoperitoneum, and a right clavicle fracture.

While hospitalized, she underwent an intramedullary (IM) nail of the left femur and open reduction internal fixation (ORIF) of the right clavicle by orthopedic surgery. Laboratory parameters were stable during the first several hospital days. Hemoglobin remained in the 7 g/dL range, and renal function tests were normal with BUN of 14–16 mg/dL and 0.9–1.0 mg/dL creatinine. Urine output averaged 0.8–1.1 mL/kg/hour and no hematuria was observed.

On approximately the 7th day of admission, subtle changes started appearing. Urine output declined to about 0.2 mL/kg/h, initially attributed to decreased oral intake. Retrospective review also showed progressively increasing systolic blood pressure rising from 110–120 mmHg to 160 mmHg. Mild elevations in serum potassium and calcium were also noted, suggesting evolving renal dysfunction.

Because the patient otherwise remained clinically stable and symptoms were nonspecific, repeat imaging was not performed earlier. However, on Day 10, she began complaining of abdominal pain associated with an acute hemoglobin drop to 5.6 g and an acute increase in creatinine to 5.1 mg/dL. Given her known G2 splenic injury, there was a concern that this was the source of her blood loss.

**Figure 2:**
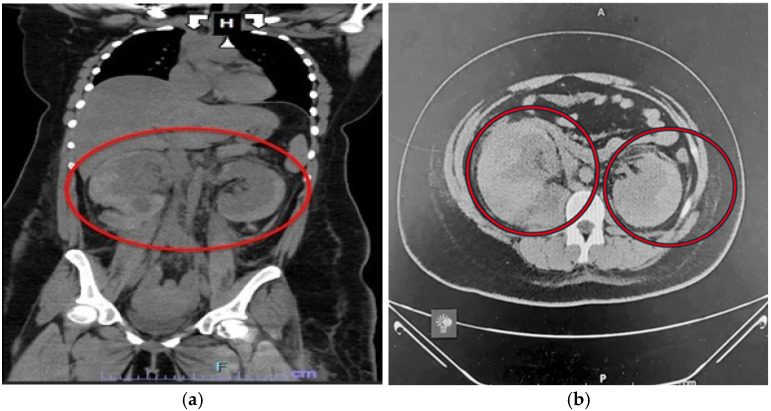
Preoperative non-contrast abdominal CT scan coronal (**a**) and axial (**b**) images, which demonstrated bilaterally enlarged perinephric hematomas (PHs). The right PH had a 4 cm diameter, and the left PH’s diameter was measured as 2 cm (ellipse).

She was resuscitated with blood products, and an abdominal computed tomographic scan without intravenous contrast (CT) scan was obtained, which demonstrated bilateral perinephric hematomas (PHs): the right PH had a 4 cm diameter, and the left PH’s diameter was measured as 2 cm (Figure 2).

**Figure 3:**
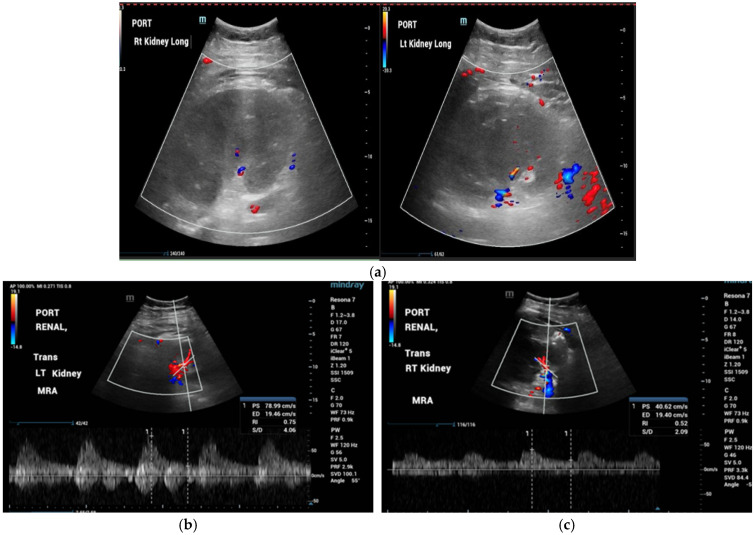
(**a**) Representative Doppler renal ultrasound demonstrating markedly reduced intrarenal perfusion with elevated resistive indices, consistent with bilateral impaired renal blood flow. (**b**) Left Kidney RI: 0.75; Left Kidney PSV/EDV: 4.06. (**c**) Right RI: 0.52; Right PSV/EDV: 2.09.

A STAT bedside renal ultrasound was performed, which demonstrated markedly reduced renal perfusion with elevated resistive indices (RIs), consistent with impaired intrarenal blood flow and decreased vascular flow in both kidneys (Figure 3a–c). The patient was taken emergently to the operating room (OR) for exploratory celiotomy for bilateral renal capsulotomy, retroperitoneal hematoma evacuation, perirenal-retroperitoneal compartment syndrome release, and a cystoscopy.

Intraoperative consultation with the Urology service was done, and a cystoscopy and cystourethrogram demonstrated normal retrograde pyelography without hydronephrosis, extravasation of contrast media, or other abnormalities.

A midline incision was done, and the abdomen was explored. Upon opening the peritoneal cavity, no free fluid or blood was noted, and the Grade II splenic injury was not bleeding. The omentum and intestinal organs extruded through the incision, suggesting abdominal hypertension due to an expanding retroperitoneal hematoma.

**Figure 4:**
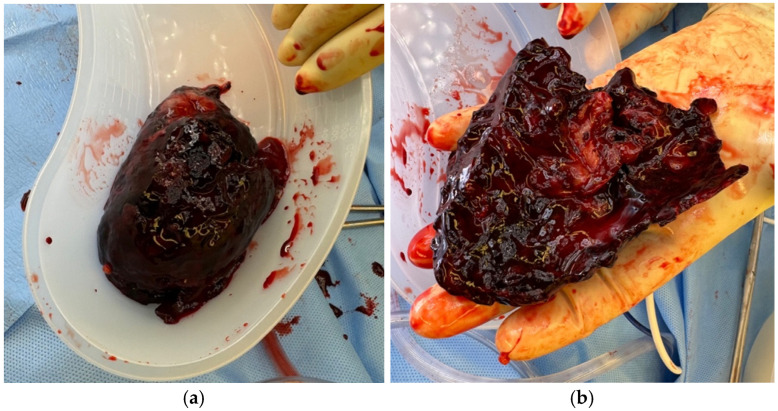
Left (**a**) and right (**b**) kidney perinephric hematomas.

We mobilized the left colon via a Mattox maneuver, exposing Gerota’s fascia. Dissection of the left renal hilum was completed, and vascular control was obtained. We then divided Gerota’s fascia and decompressed the left perinephric hematoma and evacuated the intracapsular blood clots that surrounded the kidney (Figure 4a).

**Figure 5:**
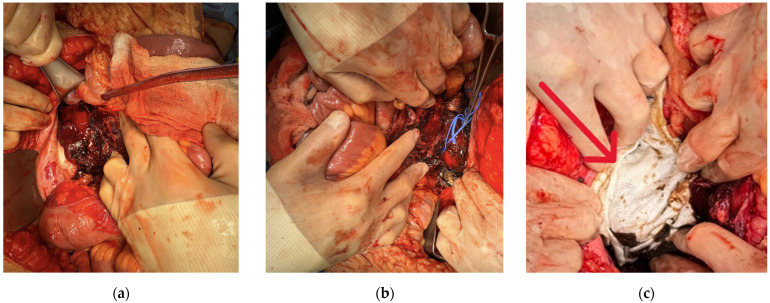
Left (**a**) and right (**b**) kidneys after capsulotomy and decompression of hematomas. (**c**) Right kidney with NuKnit wrap shown (**arrow**).

A 2 cm (Grade II) laceration on the inferior pole was identified and controlled with argon beam coagulation (ABC). The left retroperitoneum was irrigated, and all clots were suctioned. The kidney appeared healthy and was wrapped with SURGICEL NU-KNIT^®^ Absorbable Hemostatic Dressing (Ethicon, Somerville, NJ, USA) (Figure 5a).

We mobilized the right colon via a Cattel-Braasch maneuver, exposing the retroperitoneum. Proximal control of the right renal hilum was obtained, Gerota’s fascia was excised, and a right capsulotomy was performed. Again, the hematoma was evacuated, and all intracapsular clots were removed (Figure 4b). There was some bleeding from a small (Grade II) laceration of the superior pole of the kidney, which was also controlled with ABC, and the kidney was wrapped with SURGICEL NU-KNIT Absorbable Hemostatic dressing (Ethicon, Somerville, NJ, USA) (Figure 5b,c).

**Figure 6:**
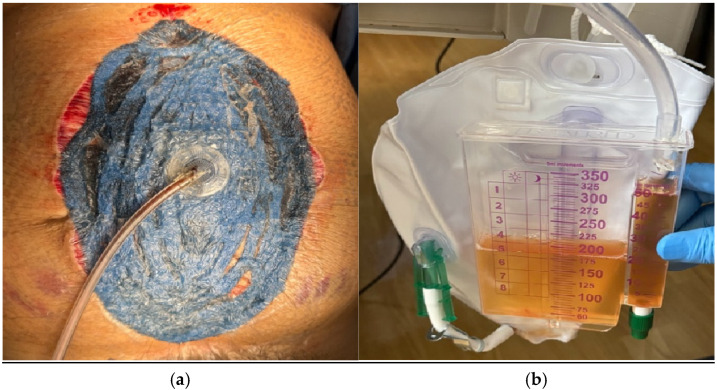
(**a**) TAC achieved with negative pressure dressing; (**b**) Brisk immediate post-operative urine production.

A careful inspection of the small bowel from the ligament of Treitz to the rectosigmoid junction, as well as the large bowel, throughout its course, was done and was unremarkable. The right and left retroperitoneal spaces appeared hemostatic. The abdomen was copiously irrigated with a hypochlorous acid solution (HOCL: Vashe^®^ Urgo Medical North America, Fort Worth, TX, USA) and placement of 3M™ AbThera™ Advance Open Abdomen Dressing (Solventum World Headquarters, Maplewood, MN, USA) was done to achieve temporary abdominal closure (TAC). The patient was scheduled for a second-look procedure in 48 h. Postoperatively, the patient’s urine output (UO) improved to 1.4 mL/kg/h, and her creatinine improved to 2.6 mg/dL along with stabilization of her hemoglobin (Figure 6a,b).

**Figure 7:**
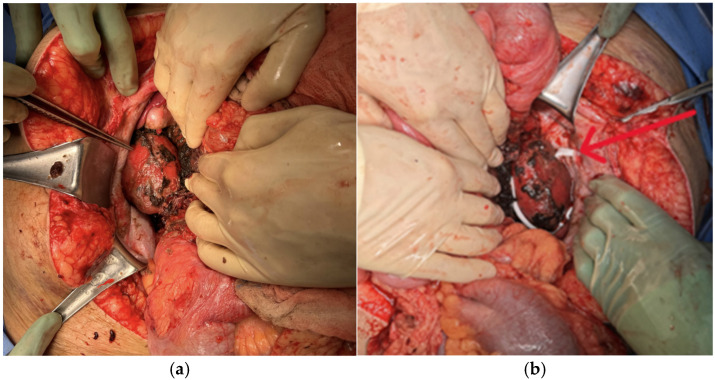
Healthy-appearing right (**a**) and left (**b**) kidneys; left JP drain placement is shown (**arrow**).

The patient returned to the OR 48 h later for re-exploration. The entirety of the bowel was run, again demonstrating no abnormalities. We inspected the right and left retroperitoneum and used HOCL solution to moisten the previously placed hemostatic gauze, which was carefully removed. There was a small venous ooze superior to the right renal hilum, which was controlled with 2 large clips and Surgicel SNoW^™^ Hemostatic Gauze (Ethicon, Raritan, NJ, USA). The kidneys appeared healthy, without any additional injuries. The right and left retroperitoneum were washed with HOCL solution, and two 10F fully perforated flat Jackson-Pratt (JP) drains were placed on each respective side (Figure 7a,b).

**Figure 8:**
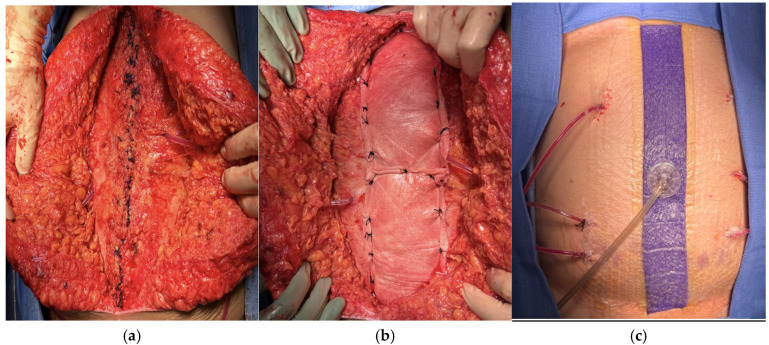
(**a**) Primary fascial closure; (**b**) Myo-cutaneous flap advancement and placement of biosynthetic mesh overlay; (**c**) placement of negative pressure dressing and subcutaneous JP drains.

The abdomen was closed with #1 interrupted Vicryl sutures. The native fascia was attenuated, so lateral subcutaneous flaps were raised bilaterally, and an onlay bio-synthetic mesh (OVITEX^®^ 2SA, 20 cm × 20 cm, OviTex 1S-P and 2S-R (TELA Bio, Malvern, PA, USA)) with 5 cm lateral overlap from the closed midline incision was utilized. The mesh was secured with #1 Nurolon simple interrupted sutures. The wound was irrigated with HOCL solution, and four subcutaneous 10F JP drains were placed (Figure 8a–c). The subcutaneous tissue was re-approximated with 3-0 Vicryl sutures, and the skin was approximated with staples. A negative pressure dressing was placed.

The patient remained intubated due to extensive bowel edema and a tight abdominal closure. The patient was chemically paralyzed and started on 25% albumin and Bumex drip for diuresis. She was then extubated 48 h later. She had an uneventful postoperative course and was subsequently discharged 7 days after her second surgery with 2 retroperitoneal drains and 2 subcutaneous drains in place.

## 3. Literature Review

### 3.1. Literature Search Strategy

A structured literature search was performed using PubMed, Scopus, and Google Scholar to identify reports of renal compartment syndrome associated with perinephric or subcapsular collections leading to renal dysfunction. Search terms included renal compartment syndrome, Page kidney, perinephric hematoma, subcapsular renal hematoma, and renal tamponade. Only peer-reviewed publications written in English were included. Titles and abstracts were screened for relevance, followed by full-text review of potentially eligible studies. Publications describing renal dysfunction secondary to subcapsular or perinephric collections were included. Using these criteria, 46 publications were identified and included in the qualitative synthesis (Table 1).

### 3.2. Summary of Table Findings

A pooled descriptive analysis of the 46 publications summarized in Table 1 identified approximately 79 reported patients with renal compartment syndrome/page-kidney physiology causing renal dysfunction. Of these, 43 involved transplanted kidneys and 36 involved native kidneys, indicating that transplant-associated disease remains the most frequently reported clinical setting. Operative decompression (open or laparoscopic hematoma evacuation/capsulotomy) was the most commonly reported treatment strategy, used in approximately 52 patients, whereas 13 patients were managed conservatively and 14 underwent other non-open interventions such as percutaneous drainage, embolization, stenting, or nephrostomy.

Partial or complete renal function recovery was reported in nearly all surgically decompressed cases and in most conservatively managed cases; however, these groups are not directly comparable because conservative management was generally reserved for more stable patients with less severe diseases.

Meaningful comparison between transplanted kidneys and traumatic native kidneys is also limited by the rarity of traumatic cases, heterogeneous reporting, and the predominance of single-patient reports. Nevertheless, the aggregate literature suggests that early decompression is most consistently associated with recovery in patients with progressive renal dysfunction and demonstrable impairment of renal perfusion.

## 4. Discussion

### 4.1. Pathophysiology of Compartment Syndromes

Acute compartment syndrome is a condition in which there is an increased pressure within a closed compartment, resulting in impaired local circulation. When there is an increase in compartmental pressure, there is a reduction in venous outflow. This causes venous pressure and venous capillary pressure to increase. If the intracompartmental pressure becomes higher than arterial pressure, a decrease in arterial inflow will also occur, leading to ischemia and eventually necrosis [1]. This is true for any compartment within the body that is surrounded by a rigid or semi-rigid structure, such as the cranium, osteo-facial areas, or the abdomen (i.e., intracranial hypertension, muscular compartment or abdominal compartment syndromes, respectively) [53,54]. Similarly, from a hydraulic point of view, the ‘renal compartment’, whose content and structure are the parenchyma and renal capsule, respectively, is similarly affected [55].

### 4.2. Renal Hemodynamics and Compartment Physiology

To function, the kidneys rely on oxygen being delivered to its tissues. The major determinants of this are renal blood flow, local tissue perfusion, and blood oxygen content. If a renal hematoma disrupts renal hemodynamics, tissue hypoxia, and acute kidney injury will ensue [56]. There have been numerous case reports in the literature describing patients presenting with renal dysfunction due to fluid collection in the renal compartment (e.g., renal allograft compartment syndrome, page kidney, or simply acute kidney injury or failure). However, to our knowledge, the diagnosis of bilateral traumatic renal compartment syndrome (BTRCS) due to trauma has not been previously described [5,57].

### 4.3. Experimental Evidence on Renal Compartment Syndrome

To prove the theory of renal compartment syndrome, an experiment was done on piglets, where they had their kidneys injected with fluid and their renal compartment pressures measured. The authors found that in healthy kidneys, pressure has a highly nonlinear dependence on the injected volume, as revealed by an exponential fit to the data (R2 = 0.92). On the contrary, a linear relation between pressure and volume is observed in decapsulated kidneys [55].

Another similar experiment was done on mice. This study found that after an ischemia–reperfusion injury, there was a significant increase in pressure values within the renal compartment in an ischemia-time-dependent manner. Without surgical treatment, a significant decrease in functional parameters was found with a considerably reduced tubular excretion rate. Surgical pressure relief was able to significantly prevent loss of tubular excretion rate and renal blood flow and preserve the integrity of renal structures [58]. Other experimental studies with similar findings were done on monkeys, dogs, and piglets [59,60,61].

### 4.4. Renal Compartment Syndrome Versus Page Kidney

Renal compartment syndrome is an acute condition in which there is an increase in pressure within the native kidney’s compartment due to peri-renal (within Gerota’s fascia) or subcapsular (within renal capsule) fluid collection. This sudden increased pressure leads to acute kidney injury, which is evidenced by an increase in serum creatinine or a reduction in urine volume [62,63]. For renal failure to ensue, there must be either bilateral involvement of both functional kidneys or involvement of a single functional kidney (i.e., having a renal allograft or having a nephrectomy of the other kidney or being born with a congenital single kidney) [64]. The proposed treatment for this condition is surgical decompression by capsulotomy.

On the other hand, page kidney, first described in 1939 by Irvine Page, is a condition in which external compression of the kidney leads to activation of the renin–angiotensin–aldosterone system (RAAS) and secondary hypertension. It is most commonly caused by chronic subcapsular hematoma. The interval between the initial causative event and the manifestation of hypertension could range between days and decades. Similarly to RCS, diagnosis is typically made using ultrasound or CT scan. Conservative management with anti-hypertensive medications, particularly those that act against the RAAS, is the mainstay [64].

### 4.5. Demographicl Distribution

The age distribution of patients developing RCS ranges from as young as 16 years to as old as 80 years, with a mean and median of ~45 years. Male patients with RCS were considerably more common than females, with a ratio of 2 to 1.

### 4.6. Etiology

The most common etiology in the development of renal compartment syndrome is dysfunction of the renal allograft (14 papers), which is still poorly understood but may be due to compression of the transplant in the limited retroperitoneal space when the anterior abdominal wall is closed. This is followed by biopsy of kidneys, especially allografts (11 papers). Other common instances include ESWL-induced trauma (6 papers), as well as cases of spontaneous bleeding (6 papers) due to chronic warfarin use, for example. Finally, accidental blunt trauma to transplanted kidneys is also reported to be the leading cause of renal compartment syndrome (2 papers).

### 4.7. Pathophysiology Behind Delayed Clinical Presentation

The delayed clinical deterioration in our patient is notable. Retroperitoneal hematomas following blunt renal trauma may initially stay stable due to tamponade within the confined retroperitoneal space. Over time, however, clot breakdown, delayed venous bleeding, or microvascular injury can lead to gradual hematoma expansion. Additionally, trauma patients often receive pharmacologic prophylaxis for venous thromboembolism during hospitalization, which may contribute to delayed enlargement of previously stable hematomas.

**Figure 9:**
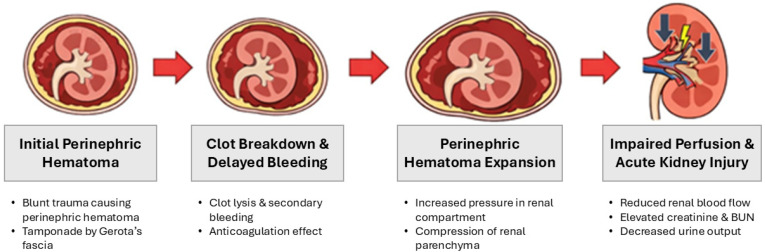
Proposed pathophysiologic mechanism of delayed renal compartment syndrome.

Progressive blood accumulation within the perinephric space enclosed by Gerota’s fascia can gradually raise renal compartment pressure until renal perfusion becomes compromised, ultimately resulting in acute kidney injury and clinical deterioration. This mechanism may explain the delayed presentation seen in our case (Figure 9).

### 4.8. Proposed Diagnostic Framework

Ultrasound is the modality of choice in almost all papers. It can detect any collections in the perinephric or subcapsular space and measure the renal flow and arterial resistance indices when Doppler is added to it. A computed tomography scan is frequently used to confirm the diagnosis. Some papers reported the use of angiography, scintigraphy, and MRI to further aid in the diagnosis. Renal function tests, which include measuring serum creatinine and urine output of the patients, are commonly used.

Based on our case and prior reports, we propose that RCS should be suspected in trauma patients with:

(1) Acute kidney injury;
(2) Perinephric/subcapsular collections on imaging;
(3) Reduced renal perfusion on Doppler ultrasound.

In retrospect, subtle clinical indicators of evolving renal dysfunction were present before the patient’s acute deterioration, including declining urine output, progressive hypertension, and electrolyte abnormalities. Recognition of these early warning signs may facilitate earlier imaging and intervention in similar cases.

**Figure 10:**
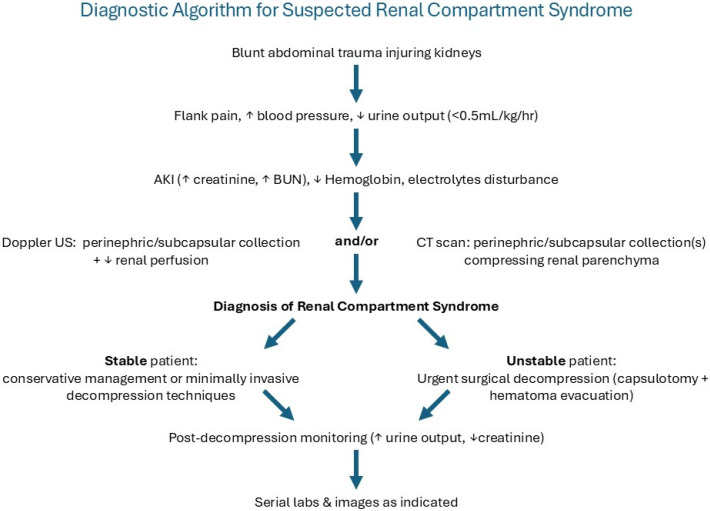
Proposed diagnostic algorithm for suspected renal compartment syndrome following trauma.

Trauma patients with acute kidney injury and perinephric or subcapsular collections on imaging should undergo prompt evaluation of renal perfusion using Doppler ultrasonography and CT imaging. Evidence of impaired renal perfusion or enlarging hematomas should prompt urgent consideration of surgical decompression. The authors suggest the following diagnostic algorithm (Figure 10).

### 4.9. Measuring Preoperative Intra-Abdominal Pressure

Although WSACS guidelines recommend intra-abdominal pressure measurement via intravesical monitoring for diagnosing intra-abdominal hypertension and abdominal compartment syndrome, it was not performed in this case before surgery [3]. The decision to proceed directly with operative exploration was based on the patient’s rapid clinical deterioration, severe acute kidney injury, and imaging findings showing enlarging bilateral perinephric hematomas with reduced renal perfusion on Doppler ultrasonography.

In such situations, delaying surgical intervention to obtain additional measurements might risk further renal ischemia and worsening organ dysfunction. During surgery, the extrusion of abdominal viscera upon entering the peritoneal cavity indicated markedly elevated intra-abdominal pressure consistent with secondary abdominal compartment physiology. However, formal IAP monitoring could be helpful in similar cases to assist with the diagnosis and management of abdominal compartment syndrome.

### 4.10. Therapeutic Intervention

Treatment for RCS patients falls into two main categories, either conservative or surgical, depending on the severity of the case and the physician’s clinical judgement. Conservative management includes blood transfusion, the administration of corticosteroids, antihypertensives, antibiotics, hemodialysis, and, on occasion, watchful waiting.

Surgical treatment ranges from percutaneous drainage or insertion of nephrostomy tubes, placement of ureteral tubes, interventional embolization, minimally invasive laparoscopy, or laparotomy. Most papers employed laparotomy to perform capsulotomy to evacuate the renal subcapsular space and to decompress the renal compartment pressure. Some authors initiated conservative management, with a subset of their patients eventually ending up in surgery due to unresolved subcapsular or perinephric hematomas and their complications. Almost all patients had partial to full recovery of their renal function, except for one case of an elderly female patient who didn’t recover and succumbed to the acute illness.

### 4.11. Role of Placing an Onlay Biosynthetic Mesh After Total Abdominal Closure

At the second-look operation, definitive primary fascial closure was achieved; however, the fascia was judged intraoperatively to be attenuated and at high risk for postoperative failure after recent open abdomen therapy [65,66].

Patients treated with open abdomen therapy are known to have a significant long-term risk of incisional hernia, and prophylactic mesh augmentation has been investigated in selected high-risk closures to reduce this risk [67,68,69,70]. For this reason, an onlay biosynthetic mesh was placed as reinforcement rather than as a bridge [71]. Nonetheless, evidence supporting this approach after TAC remains limited, and in this case, the mesh was used based on intraoperative assessment of fascial quality rather than as a routine closure component. No definitive conclusion about hernia prevention can be drawn from this single case.

### 4.12. Secondary Abdominal Compartment Syndrome in the Setting of Bilateral Renal Compartment Syndrome

An interesting finding in our case was the development of abdominal compartment syndrome, evidenced by the extrusion of abdominal organs when the abdomen was entered. Although intra-abdominal pressure was not formally measured, the clinical findings strongly suggested secondary ACS due to the bilateral renal compartment syndrome. The patient underwent damage control surgery (DCS) with temporary abdominal closure, an established strategy in trauma patients with severe hemorrhage, abdominal hypertension, or physiologic instability. This staged approach allows for rapid control of life-threatening pathology while permitting ongoing resuscitation and planned re-exploration once the patient’s physiologic status improves [3,72,73].

Unfortunately, we could not find this phenomenon described in the literature. However, a similar etiology that might possibly explain this is in the case of renal allograft compartment syndrome, where the site of raised pressure in RACS is anatomically extraperitoneal, but still intra-abdominal. Also, retroperitoneal tumors have been described as a risk factor for developing secondary ACS, which, in essence, is a space-occupying lesion, like a renal hematoma. Another possible explanation is the entity ‘poly-compartment syndrome’, where two or more anatomical compartments have elevated compartmental pressures [3,4,5].

## 5. Limitations

Several limitations should be acknowledged when interpreting this report. First, this study describes a single clinical case, which inherently limits the generalizability of the findings. While the successful management of bilateral traumatic renal compartment syndrome in this patient provides important clinical insight, broader conclusions regarding optimal diagnostic strategies or treatment algorithms cannot be drawn from a single patient.

Second, direct measurement of renal compartment pressure was not performed. The diagnosis of renal compartment syndrome in this case was based on a combination of clinical findings, radiographic evidence of bilateral perinephric hematomas, reduced renal perfusion on Doppler ultrasonography, and rapid improvement in urine output and renal function following surgical decompression.

Although this approach is consistent with previously reported cases in the literature, the absence of direct pressure measurements represents a limitation in definitively confirming the pathophysiologic mechanism.

Third, intra-abdominal pressure was not formally measured, which limits the ability to objectively confirm the presence of secondary abdominal compartment syndrome. The diagnosis in this case was inferred from intraoperative findings, including significant visceral extrusion upon entering the abdomen and the presence of large retroperitoneal hematomas.

Finally, although we performed an extensive review of the literature, the available evidence on renal compartment syndrome remains largely limited to case reports and small case series, which introduces potential publication bias and heterogeneity in reported management strategies.

Despite these limitations, this report contributes to the limited body of literature describing renal compartment syndrome involving native kidneys and highlights an extremely rare presentation of bilateral traumatic renal compartment syndrome, emphasizing the importance of early recognition and prompt surgical decompression.

## 6. Conclusions

Management of renal compartment syndrome depends on the severity of renal dysfunction, the size and location of the compressive collection, and the clinical stability of the patient. Conservative management, percutaneous drainage, or selective arterial embolization may be appropriate in carefully selected cases when renal perfusion is preserved and the hematoma remains stable. However, in patients with progressive renal dysfunction, enlarging perinephric or subcapsular hematomas, or bilateral renal involvement, urgent operative decompression should be strongly considered. In our patient, these findings prompted immediate operative exploration and bilateral capsulotomy to prevent irreversible renal injury.

In summary, this case demonstrates a patient who presented with multiple injuries, including bilateral renal subcapsular hematomas, which were due to blunt trauma from a motor vehicle collision. Doppler ultrasound and CT scan were used to diagnose the condition, along with renal function tests. We opted for open laparotomy, where capsulotomy of both kidneys was performed to evacuate the hematomas and relieve the pressure. Our patient recovered successfully and was discharged with no post-op complications. To our knowledge, this represents the first reported case applying the term “bilateral traumatic renal compartment syndrome (BTRCS)” for native kidneys following blunt trauma, which was successfully treated with bilateral surgical decompression, resulting in rapid physiological recovery.

## Figures and Tables

**Table 1:** Summary of case reports documenting renal compartment syndrome.

Ref.	Year	No. of Pt.	Age (Y)	Sex	Etiology	sCr (mg/dL)	Imaging	Treatment	Outcome (RF; Status)
[7]	1976	1	35	M	Renal allograft dysfunction	1.4	US: SC hematoma	Laparotomy for hematoma evacuation	Recovered; alive
[8]	1988	1	40	F	Renal allograft dysfunction	1.6 → 6.5 → 2.5	US: normal Tc-99m scan: normal	Corticosteroid → Laparotomy for perirenal scar dissection	Recovered w/ residual CKD; alive
[9]	1991	1	33	M	Rt renal biopsy (h/o IgA nephropathy)	1.3 → 2.7 → 2.7 → 3.8	US: large rt PN hematoma CT: an 8 × 10 × 15 cm rt PN & SC hematoma → [*after 9 months*] calcified fibrotic rt renal capsule MRI: Rt PN & SC hematoma	Conservative management	Recovered w/ residual CKD; alive
[10]	1992	1	35	M	ESWL	1.5 → 1	US: Lt PN & SC hematoma CT: RP hematoma w/ displacement of L kidney	Ureteral stent + Laparotomy for RP hematoma evacuation	Recovered; alive
[11]	1993	1	19	F	Renal allograft biopsy	7 → 2.2	Doppler US: SC hematoma w/ ↓ perfusion	Laparotomy for hematoma evacuation	Recovered w/ residual CKD; alive
[12]	2002	1	56	M	NR (h/o rt nephrectomy)	4.3 → 5.7	CT: 13 cm Lt SC hematoma Angiography: medial distortion of renal vessels	Laparotomy for hematoma evacuation	Recovered; alive
[5]	2006	11	45 *	8 M 3 F	Renal allograft dysfunction	NR	Doppler US: peri-allograft SC collection & ↑ renal arterial resistance	Laparotomy for hematoma evacuation	Recovered; alive (except 1 pt died due to unrelated malignancy)
[13]	2006	1	36	F	Spontaneous hematoma possibly due to warfarin (h/o rt nephrectomy)	1.5 → 5.7 → 1.2	Doppler US: Lt SC hematoma w/ ↓ perfusion CT: Lt SC hematoma	Reversal of anticoagulation + percutaneous drainage of hematoma	Recovered; alive
[14]	2006	1	42	F	Renal allograft dysfunction	NR	Doppler US: peri-allograft SC collection & ↑ renal arterial resistance	Laparotomy for hematoma evacuation	Recovered; alive
[15]	2007	1	46	M	ESWL	0.8 → 0.76	US: large Lt PN hematoma w/ probable rupture into RP CT: a 12 × 16 × 8 cm RP hematoma	Conservative management	Recovered; alive
[16]	2007	1	34	F	ESWL	NR	CT: bilateral PN hematomas Doppler US: no renal artery stenosis MRI: a 6.8 × 5.1 × 4.0 cm rt SC hematoma & a 5.0 × 4.0 × 2.5 cm Lt SC hematoma	Conservative management → Bilateral percutaneous drainage of hematomas → Laparoscopic drainage of hematoma	Recovered; alive
[17]	2007	1	69	M	Renal allograft biopsy	1.1 → 1.5 → 2.8 → 1.6	US: peri-allograft SC hematoma CT: a 9 × 2 cm SC hematoma	Conservative management → Laparotomy for hematoma evacuation	Recovered w/ residual CKD; alive
[18]	2008	1	64	M	Renal allograft biopsy	1.39 → 4.39 → 1.8	Doppler US: a 5.1 cm SC hematoma w/ ↓ perfusion	Laparotomy for hematoma evacuation	Recovered w/ residual CKD; alive
[19]	2008	1	71	F	Radiofrequency ablation of a 1.5 cm mass in L kidney (h/o rt nephrectomy)	4.5	CT: 8.2 × 2.4 cm Lt SC hematoma	Laparotomy for hematoma evacuation	Recovered; alive
[20]	2008	1	69	NR	Rt partial nephrectomy of RCC (h/o solitary rt kidney)	5.66→ 1.36	Doppler US: ↓ perfusion CT: rt SC hematoma	Percutaneous drainage of hematoma	Recovered; alive
[21]	2008	4	27	F	Renal allograft biopsy	7.7	Doppler US: SC hematoma w/ ↓ perfusion CT: SC hematoma + RP hematoma	Laparotomy for hematoma evacuation	Recovered; alive
			39	F		3	Doppler US: SC hematoma w/ ↓ perfusion		
			35	M		5.6	Doppler US: SC hematoma w/ ↓ perfusion CT: SC hematoma + RP hematoma		
			33	F		2.7	Doppler US: SC hematoma w/ ↓ perfusion		
[22]	2008	1	71	F	ESWL	NR	CT: 8 × 4 cm Lt SC hematoma	Conservative management	Did not recover; died
[23]	2009	1	60	M	Renal allograft dysfunction	8.6	Doppler US: [*day 0*] Urinary tract dilation → [*day 2*] 8 × 2.9 cm SC hematoma w/ ↓ perfusion Pyelogram: stenosis of ureterovesical anastomosis CT: SC hematoma	[*day 0*] Nephrostomy → [*day 2*] Percutaneous drainage of hematoma → [*day 3*] Laparotomy for hematoma evacuation	Recovered; alive
[24]	2009	1	36	M	Polyarteritis nodosa causing intrarenal micro-aneurysms	1.7 → 6.8 → 3.1	CT: 15 × 13 × 12 cm rt SC hematoma → recurrent Lt SC hematoma Angiography: multiple renal microaneurysms	Rt nephrectomy → Lt renal aneurysm embolization → IV methylprednisolone + IV cyclophosphamide + PO prednisolone + hemodialysis	NR; alive
[25]	2010	1	55	M	Renal allograft biopsy	3.5 → ~ 0.75	Doppler US: peri-allograft SC hematoma & ↑ renal arterial resistance	Laparotomy for hematoma evacuation	Recovered; alive
[26]	2010	1	61	F	Renal allograft dysfunction	5.9 → 1.2	CT: SC hematoma + proximal Rt leg DVT	IVC Filter + Laparotomy for hematoma evacuation	Recovered; alive
[27]	2010	1	16	M	Recurrent spontaneous renal hematoma (h/o congenital solitary kidney & sports-related injury)	1.7 → 1.2 → 1.4 → 1.1	US: Solitary Rt kidney w/ SC hematoma CT: SC hematoma Angiography: dysplastic capsular arteries	Repeated percutaneous drainage of hematoma & use of sclerosing agents → Dysplastic capsular artery embolization	Recovered; alive
[28]	2011	1	43	M	Spontaneous bilateral kidney hematomas possibly due to warfarin	1.8	US: bilateral hydronephrosis CT: bilateral extensive hyperdense thickening of renal & ureteral walls & high-attenuation areas	Conservative management	Recovered; alive
[29]	2011	1	45	M	Renal allograft biopsy	1.05	Doppler US: a 2 × 2 × 2 cm peri-allograft hematoma & ↑ renal arterial resistance	Laparotomy for hematoma evacuation	Recovered; alive
[30]	2012	1	46	M	Renal allograft dysfunction	6.84	Doppler US: a 3 × 9 cm peri-allograft hematoma & ↑ renal arterial resistance	Laparotomy for hematoma evacuation	Recovered; alive
[31]	2012	2	55	M	Renal allograft dysfunction	NR	DTPA: ↓ perfusion w/ no extraction Duplex US: ↓ perfusion	Laparotomy for hematoma evacuation	Recovered; alive
			61	F			Duplex US: ↓ perfusion	Laparotomy for hematoma evacuation	Recovered; alive
[32]	2012	1	35	F	NR	1.45	Doppler US: bilateral PN fluid collections w/ ↓ perfusion CT: bilateral SC fluid collections	Percutaneous drainage of fluid	Recovered; alive
[33]	2014	7	63 *	5 M 2 F	ESWL	1	CT: SC hematoma	Laparotomy for hematoma evacuation	Recovered; alive
		8	52 *	6 M 2 F		1	CT: SC hematoma	Conservative management	Recovered; alive
[34]	2015	1	67	M	Bicycle accident (h/o renal graft)	2.73 → 0.88 → 0.81	Doppler US: peri-allograft hematoma & ↑ renal arterial resistance CT: 4 cm SC hematoma	Laparotomy for hematoma evacuation	Recovered; alive
[35]	2016	1	74	F	Ureteroscopic procedure w/ biopsy causing trauma (h/o of Lt nephrectomy due to RCC)	0.9 → 6.9 → 7.9 → 1.9 → 1.2	US: Rt SC hematoma	Conservative management → hemodialysis → Percutaneous drainage of hematoma	Recovered; alive
[36]	2017	1	17	M	NR (possibly due to trauma during catheterization procedure)	1.38 → 0.9	CT: large Lt SC hematoma DTPA: ↓ perfusion in L kidney	[*day* 0] Selective Lt renal artery embolization → [*day 5*] Laparotomy for hematoma evacuation	Recovered; alive
[37]	2017	1	23	M	Early allograft dysfunction after rt kidney transplant	NR	Doppler US: no blood flow	Laparotomy for hematoma evacuation	Recovered; alive
[38]	2017	1	32	M	Bilateral idiopathic spontaneous RP hemorrhage (Wunderlich Syndrome)	8.2	US: enlarged Lt kidney & a 11.4 × 9.9 × 8.6 cm anechoic collection w/ septations CT: Lt PN collection MRI: bilateral PN hematomas CTA: 10.3 × 10.3 cm Lt SC hematoma + 3.6 × 3.1 cm rt PN & SC hematoma	Conservative management → Percutaneous drainage of a Lt PN abscess	Recovered; alive
[39]	2018	1	31	M	Renal allograft biopsy	1.6 → 4.23 → 1.89	Doppler US: peri-allograft SC hematoma w/ ↓ perfusion CT: SC hematoma	Laparotomy for hematoma evacuation	Recovered w/ residual CKD; alive
[40]	2018	1	29	M	Renal allograft biopsy	1.8 → 2.3 → 1.5	US: peri-allograft hematoma CT: 6 × 7 cm SC hematoma	Percutaneous drainage of hematoma	Recovered w/ residual CKD; alive
[41]	2018	1	66	M	Renal allograft dysfunction	6 → 1.7	Doppler US: ↓ perfusion in upper pole of allograft CT: small renal cyst at middle to upper pole of allograft	Laparotomy for hematoma evacuation	Recovered w/ residual CKD; alive
[42]	2019	1	80	M	Percutaneous radiofrequency ablation of a renal tumor (h/o left nephrectomy)	2.1→ 4.33 → 6.4 → 2.3	Doppler US: rt SC hematoma w/ ↓ perfusion	Percutaneous drainage of hematoma	Recovered w/ residual CKD; alive
[43]	2020	1	49	M	Renal allograft dysfunction		Duplex US: peri-allograft SC hematoma & ↑ renal arterial resistance CT: a 1.7 cm peri-allograft SC hematoma	Laparotomy for hematoma evacuation	Recovered; alive
[44]	2020	2	36	M	Renal allograft biopsy	4.9	Doppler US: a 6.7 × 2.1 × 7.1 cm peri-allograft SC hematoma & ↑ renal arterial resistance	Laparotomy for hematoma evacuation	Recovered; alive
			68	M		7.4	Doppler US: a 1.7 × 1.3 cm arteriovenous fistula in graft upper pole → a 9.3 × 3.3 × 4 cm peri-allograft SC hematoma & ↑ renal arterial resistance	Percutaneous drainage of hematoma → Laparotomy for hematoma evacuation	
[45]	2020	1	50	F	Spontaneous bilateral renal hematoma due to microscopic polyangiitis	1.9	CT: [*Day 0*] Lt PN hematoma → [*day 2*] bilateral PN hematomas	Bilateral percutaneous drainage of hematomas + methylprednisolone + cyclophosphamide.	NR
[46]	2021	4	24	M	Renal allograft dysfunction	3.7	Doppler US: 450 mL peri-allograft collection CT: large peri-allograft hematoma	Laparotomy for hematoma evacuation	Recovered; alive
			18	M		1.7	Doppler US: ~2 L infraumbilical collection CT: peri-allograft collection & kinking of ureter (lymphocele)	Laparoscopic fenestration of lymphocele + opening marsupialization	Recovered; alive
			25	M		4.8	Doppler US: [*day 3*] ↓ perfusion → [*day 7*] ~2 L peri-allograft collection CT: large peri-allograft hematoma	Stenting for transplant artery stenosis + Aspiration & pigtail catheter drainage for hematoma	Recovered; alive
			18	F		2	Doppler US: ~2 L peri-allograft & pelvic collection; normal perfusion CT: pelvic collection compressing lower pole of graft (lymphocele)	Laparoscopic fenestration of lymphocele + opening marsupialization	Recovered; alive
[47]	2022	1	16	M	Renal allograft dysfunction	2.97 → 1.57	Doppler US: peri-allograft SC hematoma w/ ↓ perfusion	Laparotomy for hematoma evacuation	Recovered w/ residual CKD; alive
[48]	2022	1	56	M	Renal allograft dysfunction	6.03 → 0.98	Doppler US: 2.8 cm peri-allograft SC hematoma w/ ↓ perfusion	Laparotomy for hematoma evacuation	Recovered; alive
[49]	2022	1	73	F	Polyarteritis nodosa causing intrarenal micro-aneurysms	5.5	CT: bilateral SC hematomas (rt:10.8 × 10 × 18 cm; Lt: 14.4 × 13.3 × 15 cm) Angiography: minor aneurysms in both renal arteries	IV methylprednisolone + IV cyclophosphamide	Recovered; alive
[50]	2022	1	Late 70s	M	MVC causing blunt abdominal trauma (h/o renal allograft)	NR	Doppler US: SC hematoma w/ ↓ perfusion CT: multiple fractures + large PN & RP hematoma	Laparotomy for hematoma evacuation	Recovered; alive
[51]	2023	1	48	F	Hydronephrosis-induced intrarenal pressure	4.01	US: bilateral hydronephrosis & PN fluid collection CT: bilateral hydronephrosis & PN fluid collection	Conservative management → [*day 8*] bilateral ureteral stents → [*day 21*] Percutaneous drainage of hematoma	Recovered; alive
[52]	2023	1	40	M	ESWL on rt ureter + Spontaneous Lt renal hematoma	0.97 → 1.48 → 3.8 → 8.97 → 4.22	CT: [*Day 0*] normal → [*day 4*] multiple hemorrhages & hypodense lesions in rt kidney + bladder clot → [*day 7*] hemorrhage & pneumatization in rt kidney & blood in bladder → [*day 13*] bleeding in both kidneys US: gradually increasing Lt PN hematoma	[*day 4*] Bladder clot removal & bilateral ureteral double-J tube placement & rt renal artery embolization → [*day 7*] repeat rt renal artery embolization → [*day 13–32*] conservative management → [*day 33*] Lt renal artery embolization	Recovered w/ residual CKD; alive on lifelong hemodialysis

**Pt:** patient(s); **Y**: years; **M:** male; **F**: female; **NR:** not reported; **H/o**: history of; **Rt**: right; **ESWL**: extracorporeal shock wave lithotripsy; **Lt**: left; **RCC**: renal cell carcinoma; **w/**: with; **RP**: retroperitoneal; **MVC:** motor vehicle collision; **sCr**: serum creatinine; **US**: ultrasound; **SC**: subcapsular; **Tc-99m**: Technetium-99m; **CT**: computed tomography; ↓: decreased/ reduced; **↑:** increased/ elevated; **MRI**: magnetic resonance imaging; **PN**: perinephric; **DTPA:** diethylenetriaminepentaacetic acid; **DVT:** deep vein thrombosis; **CTA**: computed tomography angiography; **IV**: intravenous; **PO**: per os; **IVC:** inferior vena cava; **RF**: renal function; **CKD**: chronic kidney disease. * This is the median age of subjects.

## Data Availability

All relevant data supporting the findings of this study are included within the article.

## Abbreviations

The following abbreviations are used in this manuscript:

ACS	Acute Compartment Syndrome
ABC	Argon Beam Coagulation
AKI	Acute Kidney Injury
BTRCS	Bilateral Traumatic Renal Compartment Syndrome
CKD	Chronic Kidney Disease
CT	Computed Tomography
CTAPiv	Computed Tomography of the Abdomen & Pelvis with Intravenous Contrast
CTA	Computed Tomography Angiography
DTPA	Diethylenetriaminepentaacetic Acid
DVT	Deep Vein Thrombosis
ESWL	Extracorporeal Shock Wave Lithotripsy
GCS	Glasgow Coma Scale
HOCL	Hypochlorous Acid
IM	Intramedullary
IV	Intravenous
IVC	Inferior Vena Cava
JP	Jackson–Pratt
MVC	Motor Vehicle Collision
NR	Not Reported
OR	Operating Room
ORIF	Open Reduction and Internal Fixation
PH	Perinephric Hematoma
PN	Perinephric
PO	Per Os (by mouth)
PSV/EDV	Peak Systolic Velocity/End-Diastolic Velocity
RACS	Renal Allograft Compartment Syndrome
RCC	Renal Cell Carcinoma
RCS	Renal Compartment Syndrome
RF	Renal Function
RI	Resistive Index
RP	Retroperitoneal
SC	Subcapsular
sCr	Serum Creatinine
TAC	Temporary Abdominal Closure
Tc-99m	Technetium-99m
US	Ultrasound
UO	Urine Output
WSACS	World Society of the Abdominal Compartment Syndrome
